# Granulomatosis With Polyangiitis (GPA) Presenting With Painless Scleritis and Ocular Hypertension: Case Report

**DOI:** 10.1155/crii/6631904

**Published:** 2025-11-18

**Authors:** Khaled A. Elubous, Hady Saheb, Karin M. Oliver

**Affiliations:** ^1^Department of Special Surgeries, Faculty of Medicine, The Hashemite University, Zarqa, Jordan; ^2^Department of Ophthalmology and Visual Sciences, McGill University, Montreal, Canada

**Keywords:** episcleral venous pressure, glaucoma, granulomatosis with polyangiitis, intraocular pressure, muffled hearing, ocular hypertension, Schlemm's canal, scleritis, vasculitis

## Abstract

This case report describes a 67-year-old male who presented with a 2-month history of painless left eye redness and muffled hearing. Ophthalmologic examination revealed elevated intraocular pressure (IOP) and significant conjunctival injection without associated pain or visual disturbance. Blood was observed in Schlemm's canal (SC), and a thorough investigation for elevated episcleral venous pressure (EVP) was performed. Imaging, including magnetic resonance imaging (MRI) and cerebral angiogram, was unremarkable. A rheumatologic workup led to the diagnosis of granulomatosis with polyangiitis (GPA). The patient's ocular symptoms improved significantly with systemic steroid treatment. He was subsequently managed with rituximab and avacopan as per standard GPA therapy. This case highlights the importance of considering vasculitis in patients presenting with unexplained elevation of EVP, and painless scleritis, particularly when there are also extraocular complaints.

## 1. Introduction

Blood in Schlemm's canal (SC) typically results from two primary mechanisms: hypotony or increased resistance in the episcleral venous system. The latter, characterized by elevated episcleral venous pressure (EVP), may arise from orbital congestion or venous abnormalities [1]. Elevated EVP is an uncommon but important cause of secondary glaucoma, and it requires careful evaluation to determine the underlying etiology. The differential diagnosis of elevated EVP includes conditions such as venous congestion, arteriovenous fistulas, and idiopathic causes [2]. While less common, venous congestion can also be associated with vasculitis involving the episcleral vessels or orbital veins [3].

Granulomatosis with polyangiitis (GPA), formerly known as Wegener's granulomatosis, is a systemic vasculitis affecting small- and medium-sized vessels, commonly involving the respiratory tract and kidneys. Ocular manifestations are frequent, with episcleritis, conjunctivitis, scleritis, and orbital inflammation being the most prevalent [4].

In this report, we describe a rare presentation of GPA where the patient exhibited painless scleritis, elevated intraocular pressure (IOP) and blood in SC, in addition to muffled hearing. These ocular findings were key to initiating further investigations, ultimately leading to a diagnosis of GPA and successful treatment with systemic corticosteroid. This case underscores the importance of considering systemic vasculitis in patients presenting with signs of unexplained elevation of EVP or painless scleritis particularly, when associated with other systemic signs.

## 2. Case Presentation

A 67-year-old male with a history of hypertension presented with a 2-month history of left eye redness, muffled hearing, and sinusitis. The patient denied ocular pain or changes in vision.

On ophthalmologic examination, the patient's visual acuity was 20/20 in both eyes, his IOP was 32 OD and 46 OS. Bilateral conjunctival injection was noted, with quiet anterior chambers. Gonioscopy revealed open angles without initial evidence of blood in SC. Fundus examination showed a disc hemorrhage in the left eye. Computed tomography (CT) and CT angiography (CTA) of the head were unremarkable, except for partial opacification of the left mastoid air cells. Antiglaucoma medication was initiated including dorzolamide/timolol and brimonidine twice daily OU, as well as oral acetazolamide (500 mg twice daily).

**Figure 1:**
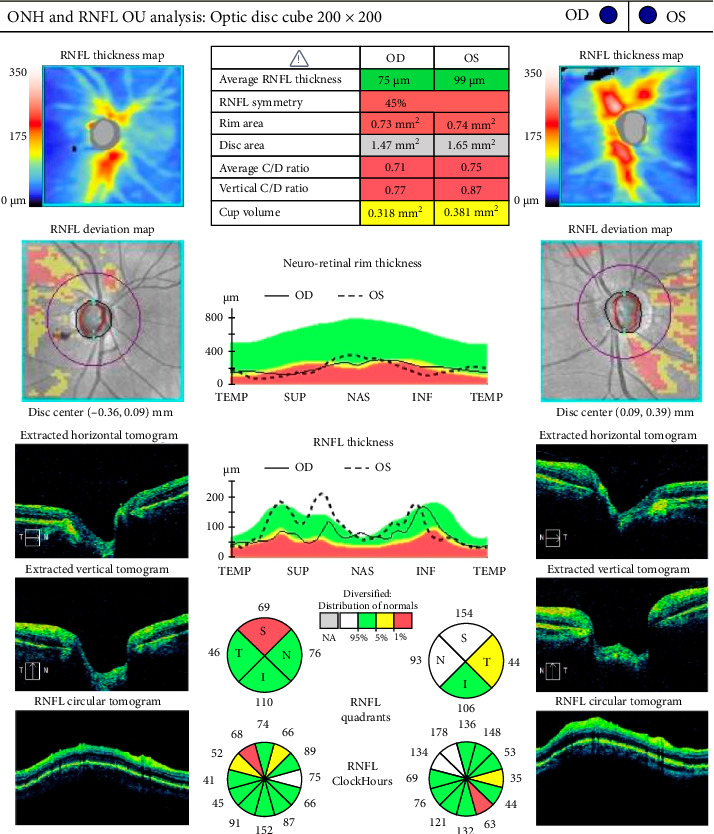
Optical coherence tomography (OCT) of the retinal nerve fiber layer (RNFL) reveals thickening of the superior RNFL bundle in the left eye, associated with disc edema and hemorrhage. The right eye shows RNFL thinning.

**Figure 2:**
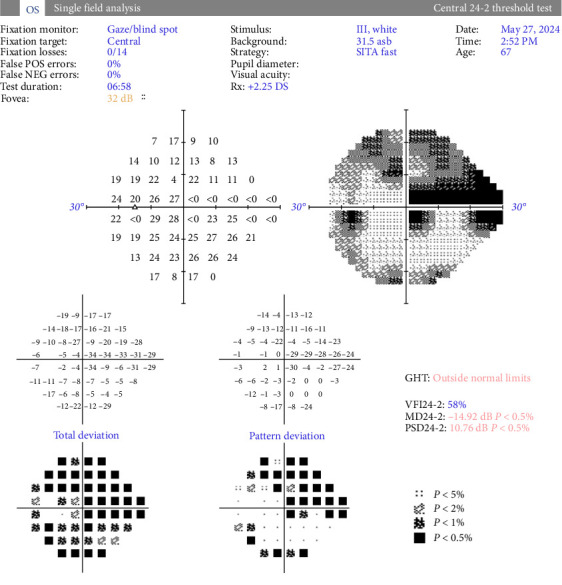
Standard automated perimetry of the left eye showing superotemporal and central defects.

At follow-up, 1 week later, the IOP in the left eye remained elevated at 22 OD, 25 OS mmHg, on the presicribed antiglaucoma medication, and blood was observed in SC. Optical coherence tomography (OCT) of the optic disc showed thickened retinal nerve fiber layer (RNFL) in the left eye (see Figure 1). Visual field testing revealed a central field defect (see Figure 2). These findings necessitated an urgent investigations to rule out arteriovenous fistula.

Magnetic resonance angiography (MRA) and venography (MRV) showed no evidence of a fistula. Despite negative imaging, a cerebral angiogram was performed and was also unremarkable. Rheumatologic and ENT evaluations were initiated, given the constellation of ocular, auditory, and sinus symptoms.

Laboratory findings revealed elevated anti-PR3 levels (>200 RU/mL), positive ANA (1:80), and elevated IgG4 levels. Anti-MPO was negative. Renal function tests indicated proteinuria and elevated creatinine. Based on clinical and laboratory findings, the patient was diagnosed with GPA.

The patient was started on systemic corticosteroids (prednisone 50 mg daily) with a tapering schedule, alongside vitamin D supplementation, resulting in appreciable improvement of symptoms and IOP. Rituximab and Avacopan, a selective C5a receptor antagonist, were initiated under the Advocate trial protocol. Upon initial of PO prednisone, the oral NSAIDS were discontinued. Over the course of 6 months, the patient's IOP and conjunctival redness improved, ranging from 10 to 17, and systemic control of GPA was achieved.

## 3. Discussion

The differential diagnosis for elevated EVP includes arteriovenous fistulas, such as carotid-cavernous fistulas, dural arteriovenous malformations, and venous obstructions like superior vena cava syndrome or orbital tumors [2]. These conditions can cause impaired venous drainage from the orbit, resulting in elevated EVP and secondary glaucoma [5]. When structural abnormalities are absent, idiopathic conditions or thyroid eye disease may also be considered [6]. Diagnostic imaging plays a pivotal role in the evaluation of elevated EVP. CT and magnetic resonance imaging (MRI) are valuable tools in detecting any orbital masses or vascular anomalies that might be contributing to venous congestion. MRA and CT angiography (CTA) are particularly useful for identifying arteriovenous fistulas, as they can detect fistulas, proptosis, or superior ophthalmic vein enlargement [7]. In highly suspicious cases where noninvasive imaging fails to identify an abnormality, cerebral angiography remains the gold standard for diagnosing arteriovenous malformations or fistulas [8]. In this case, despite the lack of findings on MRI and CTA, a cerebral angiogram was performed due to high clinical suspicion, but the results were unremarkable, ruling out an arteriovenous fistula.

When no structural cause of elevated EVP is identified, systemic inflammatory conditions, such as vasculitis, should be considered. Among these, vasculitides like GPA can manifest with ocular symptoms as a primary presentation. GPA is a necrotizing granulomatous vasculitis that typically affects the respiratory tract and kidneys, but ocular involvement is also common. Ocular manifestations are present in up to 60% of GPA patients. These include episcleritis, scleritis, uveitis, and orbital inflammation, with episcleritis being the most common ocular finding [4].

Scleritis is usually painful; however, in certain conditions like diffuse anterior scleritis, scleromalacia perforans, or in patients receiving immunosuppressive therapy, it can present without pain. Episcleritis, on the other hand, often manifests as painless diffuse redness, which can make differentiation from scleritis challenging. The use of 2.5% phenylephrine aids in diagnosis, as it causes blanching in episcleritis but not in scleritis. Scleritis may also leave a bluish discoloration of the sclera following inflammation, a feature absent in episcleritis. Glaucoma is more frequently observed in cases of scleritis, where it has been reported in up to 42% of patients with certain subtypes [9]. Glaucoma can result from different mechanisms, including open angle, closed angle, or neovascular glaucoma [10].

The precise mechanism for glaucoma secondary to elevated EVP remains unclear, but anterior segment fluorescein angiography has revealed rapid blood flow through engorged episcleral veins in affected patients [11]. This venous congestion leads to increased distal outflow resistance, reducing the pressure gradient between IOP and EVP, ultimately resulting in elevated IOP [12].

The simultaneous involvement of the sinuses and ocular structures further supports the diagnosis of GPA. Sinonasal inflammation is frequently seen in patients with GPA and can manifest alongside ocular symptoms such as conjunctival redness or episcleritis [13]. Importantly, in addition to ocular and sinonasal features, the patient demonstrated renal involvement with proteinuria and elevated creatinine. In this case, the patient's two-month history of left eye redness, sinusitis, and muffled hearing raised suspicion for a systemic vasculitic process. The diagnostic workup revealed elevated anti-PR3 levels, consistent with GPA.

The management of GPA requires prompt initiation of systemic immunosuppression to prevent progression and irreversible damage. Treatment includes high-dose corticosteroids in combination with immunosuppressive agents like cyclophosphamide or methotrexate. Rituximab, a monoclonal anti-CD20 antibody, can also achieve good results [14]. Recently, Avacopan, a selective C5a receptor antagonist, has emerged as an additional treatment option. The Advocate trial demonstrated that Avacopan was noninferior to prednisone tapering in achieving remission at 26 weeks and superior for sustained remission at 52 weeks [15]. Importantly, Avacopan helps mitigate the side effects associated with long-term glucocorticoid use [16]. In this patient, the initiation of systemic corticosteroids led to significant improvement in ocular symptoms and systemic control of the disease. After few days of treatment, IOP normalized, and conjunctival redness resolved, highlighting the drug's efficacy in controlling both ocular and systemic inflammation.

In conclusion, this case emphasizes the need to consider systemic vasculitis, particularly GPA, in differential diagnosis of elevated EVP or painless scleritis. The simultaneous involvement of ocular and sinonasal structures is a key diagnostic clue, and early recognition and treatment conclusion, can improve outcomes and prevent further complications.

## Data Availability

Data supporting this case report are available from the corresponding author upon reasonable request.

## References

[1] Wutthayakorn W., Meethongkam K., Pukrushpan P., Chansangpetch S. (2020). Intraocular Pressure Elevation Associated With Blood in Schlemm’s Canal After Strabismus Surgery. *American Journal of Ophthalmology Case Reports*.

[2] Sun C. Q., Medert C. M., Chang T. C. (2020). Idiopathic Elevated Episcleral Venous Pressure in a Teenager. *American Journal of Ophthalmology Case Reports*.

[3] Schultz H., Alexis D., Higginbotham E. J., Albert D. M., Miller J. W., Azar D. T., Young L. H. (2022). Glaucoma Associated with Episcleral Venous Pressure. *Albert and Jakobiec’s Principles and Practice of Ophthalmology*.

[4] Moin K. A., Yeakle M. M., Parrill A. M. (2023). Ocular and Orbital Manifestations of Granulomatosis With Polyangiitis: A Systematic Review of Published Cases. *Romanian Journal of Ophthalmology*.

[5] Cymbor M., Knapp E., Carlin F. (2013). Idiopathic Elevated Episcleral Venous Pressure With Secondary Glaucoma. *Optometry and Vision Science*.

[6] Betzler B. K., Young S. M., Sundar G. (2022). Intraocular Pressure and Glaucoma in Thyroid Eye Disease. *Ophthalmic Plastic & Reconstructive Surgery*.

[7] Chen C. C., Chang P. C., Shy C. G., Chen W. S., Hung H. C. (2005). CT Angiography and MR Angiography in the Evaluation of Carotid Cavernous Sinus Fistula Prior to Embolization: A Comparison of Techniques. *American Journal of Neuroradiology*.

[8] González S. B., Busquets J. C., Figueiras R. G. (2009). Imaging Arteriovenous Fistulas. *American Journal of Roentgenology*.

[9] Dutta Majumder P., Agarwal S., Shah M. (2024). Necrotizing Scleritis: A Review. *Ocular Immunology and Inflammation*.

[10] de la Maza M. S., Tauber J., Foster C. S. (2012). Clinical Considerations of Episcleritis and Scleritis. *The Sclera*.

[11] Nieuwenhuizen J., Watson P. G., Emmanouilidis-van der Spek K., Keunen J. E., Jager M. J. (2003). The Value of Combining Anterior Segment Fluorescein Angiography With Indocyanine Green Angiography in Scleral Inflammation. *Ophthalmology*.

[12] Ichhpujani P. (2022). Commentary: Idiopathic Elevated Episcleral Venous Pressure - Tension With Twisted Tortuousity. *Indian Journal of Ophthalmology*.

[13] Junek M. L., Zhao L., Garner S. (2023). Ocular Manifestations of ANCA-Associated Vasculitis. *Rheumatology*.

[14] Sfiniadaki E., Tsiara I., Theodossiadis P., Chatziralli I. (2019). Ocular Manifestations of Granulomatosis With Polyangiitis: A Review of the Literature. *Ophthalmology and Therapy*.

[15] Jayne D. R. W., Merkel P. A., Schall T. J., Bekker P. (2021). Avacopan for the Treatment of ANCA-Associated Vasculitis. *New England Journal of Medicine*.

[16] Cortazar F. B., Niles J. L., Jayne D. R. W. (2023). Renal Recovery for Patients With ANCA-Associated Vasculitis and Low eGFR in the ADVOCATE Trial of Avacopan. *Kidney International Reports*.

